# Design and Simulation Test of Non-Contact Voltage Sensor

**DOI:** 10.3390/s25103118

**Published:** 2025-05-15

**Authors:** Haojie Peng, Hongwei Liu, Kuo Shang, Gaoyue Li, Liping Zhao

**Affiliations:** 1 College of Electrical and Electronic Engineering, North China Electric Power University, Beijing 102206, China; hjpeng@ncepu.edu.cn (H.P.); 50102880@ncepu.edu.cn (L.Z.); 2 State Grid Liaoning Electric Power Research Institute Co., Ltd., Shenyang 110002, China; 13141353636@sina.cn; 3 China Electric Power Research Institute Co., Ltd., Beijing 102206, China; lgyoutlook@163.com

**Keywords:** non-contact voltage measurement, electric field coupling, micro sensor

## Abstract

The miniaturization of sensors and non-contact measurement techniques is currently at the forefront of smart grid development. This paper proposes a miniature voltage sensor whose size is significantly reduced while maintaining large bandwidth and high linearity. To minimize the impact of environmental factors on measurement accuracy, a differential structure is utilized to optimize the sensor. The sensor is designed with a dual-channel measurement mode for both high-frequency and power-frequency signals, addressing issues of signal refraction and reflection due to impedance mismatch. COMSOL Multiphysics 6.2 is employed to simulate the sensor’s structural design and placement. Moreover, the experimental analysis of key parameters, such as parallel resistance and capacitance, identifies the optimal parameter combination for low-voltage distribution lines and cables of 10 kV and below. Experiments show that the voltage sensor’s bandwidth ranges from 30 Hz–200 kHz when measured through a frequency response analyzer. Finally, through the measurement carried out on the overhead line and cable, we evaluate the linearity of the sensor according to the experimental data. Specifically, the nonlinear errors of the voltage measurement for the overhead line and cable are 0.62% and 0.57%, respectively.

## 1. Introduction

With the construction of smart grids and the installation of large-scale smart instruments [1], the wide application of sensor networks facilitates the real-time monitoring of electric energy from the generation side to the client side. It plays an important role in the status monitoring of the distribution network [2,3]. Therefore, obtaining stable and reliable electric field measurement parameters through advanced sensing technology is important for constructing a robust distribution network, achieving precise fault diagnosis and location, and ensuring the safe operation of various types of equipment [4].

At present, the commonly used device for voltage measurement is the Voltage Transformer (VT), which is divided into the Potential Transformer (PT) and the Capacitor Voltage Transformer (CVT). The PT has many advantages such as simple structure, facilitation, proficient production process, and high measurement accuracy. However, the PT also has disadvantages such as large volume, complex insulation structure, small measurement bandwidth, and bad ferromagnetic saturation, leading to serious over-voltage [5]. The CVT has the advantages of insulated structure simplicity, high cost-effectiveness, wide dynamic measurement range, and good maintainability [6]. However, similar to the PT, the size of the CVT is also large. Moreover, the CVT critically depends on voltage divider capacitors for operation, and it must incorporate dedicated ferromagnetic resonance suppression circuits.

In recent years, Shen Gao has proposed a non-contact electric field-coupled D-dot voltage sensor model [7,8], which exhibits broad application prospects due to its small size, good transient performance, and non-reliance on wired connectivity. Since the D-dot voltage sensor has no iron core saturation and does not experience secondary ferromagnetic resonance, it is easy to maintain due to its simple structure. However, external environment variations can significantly impact the capacitance between probes and the power line, leading to the waveform distortion of the integrating circuit and further inaccurate measurements [9]. To address this challenge, a differential probe design consisting of two parallel electrode plates is proposed [9,10,11]. This design offers enhanced resistance to external disturbances and thus minimizes measurement errors. Nevertheless, these sensors require back-end integration circuits to convert the current signal into a voltage signal, which may result in a loss of frequency band performance [12,13]. Zhao Pengcheng et al. optimized the design of sensors using differential structures [14,15,16,17], reducing the impact of the environment while eliminating the integrating circuit. However, the sensor cannot overcome the impedance mismatching problem during signal transmission, leading to its low measurement bandwidth. Moreover, the refraction and reflection of the high-frequency response will result in measurement deviation. Wei He et al. [18,19,20] proposed a differential D-dot sensor model with a symmetrical structure to address the waveform distortion problem caused by the irregular shape of the electrode plate and the asymmetry of the sensor placement. The waveform quality of the sensor is improved by increasing the equivalent square of the electrode relative to the live conductor. Considering the inconvenience caused by the fully enclosed structure of the sensor during installation, an open–close structure has been proposed to improve the sensor [21]. Regarding the issue of impedance mismatching, Wu Xutao et al. refined the sensor by replacing inter-electrode capacitors with multi-layer ceramic capacitors, which expands the measurement bandwidth to 30 kHz [22] and achieves the accurate measurement of the operating voltage of switch-type devices and the transient voltage of arc discharge [23].

The above voltage measurement methods have their advantages and specific drawbacks. Therefore, there is an urgent need for a non-contact, wide-bandwidth, and small-size voltage sensor that can provide voltage monitoring to the most intelligent electrical equipment.

## 2. Principles of the Sensor

**Figure 1:**
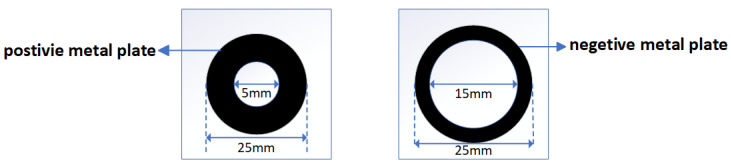
Positive and negative plate design of the differential structure sensor. (**Left**): sensor positive plate; (**right**): sensor negative plate.

This article adopts a differential structure to solve the problems of low conversion ratio and large measurement error due to the environmental impact of unipolar plate sensors. The design diagram of the differential sensors is presented in Figure 1. The outer diameter of the positive plate is 25 mm, and the inner diameter is 5 mm. The outer diameter of the negative plate is also 25 mm, and the inner diameter is 15 mm. The spacing *w* between the two plates is 1.2 cm.

**Figure 2:**
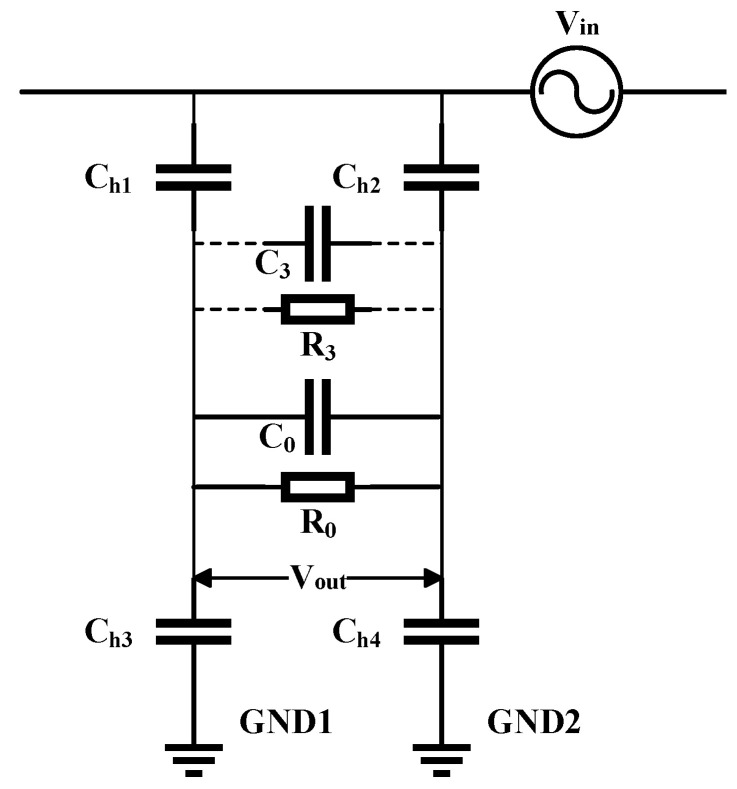
The equivalent circuit diagram of the differential structure sensor.

The equivalent circuit diagram of the differential sensor is shown in Figure 2, where $C_{h1}$ represents the coupling capacitance between the live conductor and the positive electrode plate; $C_{h2}$ represents the coupling capacitance between the live conductor and the negative electrode plate; $C_{h3}$ refers to the coupling capacitance between the positive electrode plate and the earth; $C_{h4}$ represents the coupling capacitance between the negative electrode plate and the earth; $C_{0}$ denotes the coupling capacitance between the positive and negative plates; $R_{0}$ represents the load resistance; $C_{3}$ represents the parallel capacitance between the positive and negative plates; and $R_{3}$ denotes the parallel resistance between the positive and negative plates. The transfer function of the differential sensor is described as follows:

(1) $$H\left(s\right)=\frac{V_{out}}{V_{in}}=\frac{sR_{0}C_{1}}{1+sR_{0}C_{2}},$$

(2) $$C_{1}=\frac{C_{h1}C_{h4}-C_{h2}C_{h3}}{C_{h1}+C_{h2}+C_{h3}+C_{h4}},$$

(3) $$C_{2}=\frac{\left(C_{h1}+C_{h3}\right)\left(C_{h2}+C_{h4}\right)}{C_{h1}+C_{h2}+C_{h3}+C_{h4}}+C_{0}.$$

(1) indicates that the transformation ratio of the sensor is not constant. When the term $sR_{0}C_{2}\gg 1$, we have

(4) $$H\left(s\right)=\frac{V_{out}}{V_{in}}\approx \frac{sR_{0}C_{1}}{sR_{0}C_{2}}=\frac{C_{1}}{C_{2}}.$$

Now, the transfer function is constant, which means that the sensor ratio remains constant. Through experimental measurement, the order of magnitude of $C_{h1}$, $C_{h2}$, as well as $C_{h3},C_{h4}$, is $10^{-12}$, while the order of magnitude of $C_{0}$ is $10^{-9}$. Therefore, the value of $C_{2}$ is determined by the coupling capacitance $C_{0}$ between the two metal plates, and the condition $sR_{0}C_{2}\gg 1$ can be transformed as $sR_{0}C_{0}\gg 1$ or, equivalently,

(5) $$R_{0}C_{0}\gg \frac{1}{\omega }.$$

The dimensions of the two plates and the distance between them have been presented at the beginning of this section. Specifically, in the measurement process, we found that $C_{0}$ ranges from 1.2 nF to 1.5 nF. When the AC power-frequency is 50 Hz and the load resistance is $10^{6}$ Ω, then $\omega R_{0}C_{0}=0.377<1$, which does not meet the requirement in (5). Therefore, this study incorporates a capacitor $C_{3}$ in the middle of the positive and negative plates, which is connected in parallel to $C_{0}$ to stabilize the sensor gain. In this case, the sensor meets the following conditions:

(6) $$R_{0}\left(C_{0}+C_{3}\right)\gg \frac{1}{\omega }.$$

Through experimental trials, connecting a 2.2 nF capacitor $C_{3}$ in parallel between the sensor plates can stabilize the sensor gain for both power-frequency and high-frequency voltage measurements, thereby accurately reflecting the measured voltage amplitude.

## 3. Sensor Design

In this section, we will present the details of the proposed sensor design. It is worth mentioning that this article utilizes epoxy resin as the insulation material between the positive and negative electrodes due to its high dielectric constant, low density, and low cost.

### 3.1. Factors Affecting the Transformation Ratio

**Figure 3:**
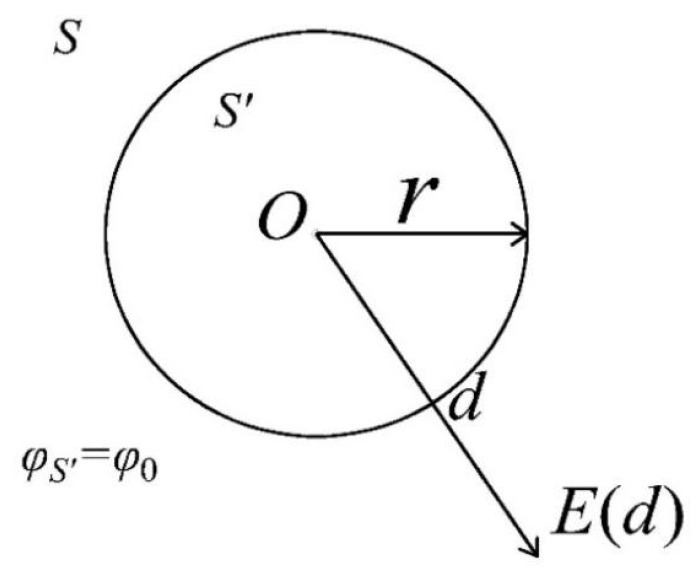
Diagram of the cylindrical conductor.

**Figure 4:**
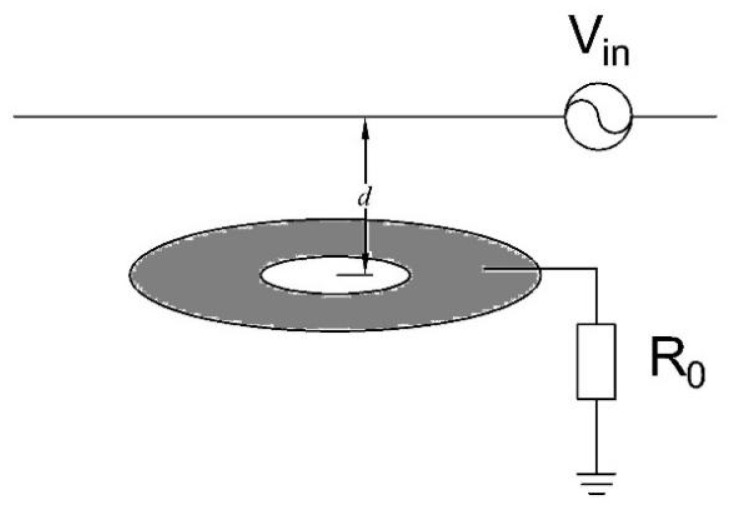
Basic model of non-contact electric field measurement.

Overhead transmission lines and cables can be regarded as cylindrical wires in a limited length range. The diagram of the cylindrical conductor is shown in Figure 3. Suppose $\varphi_{0}$ refers to the electric potential of the live conductor; the electric field intensity at a certain point is given by

(7) $$E\left(d\right)=\epsilon_{0}f\left(r,d\right)\varphi_{0},$$

where $\epsilon_{0}$ refers to the dielectric constant of the air, *r* represents the radius of the live conductor, and *d* represents the distance from a point to the live conductor. Furthermore, since d≫r, f(r,d) can be simplified as f(d). Figure 4 shows the basic model of non-contact electric field measurement. The amount of induced charge in the plate *q* can be obtained from Gauss’s theorem:

(8) $$q=∯\epsilon_{0}E\left(d,t\right)dA,$$

By further derivation and taking (7) into account, the induced voltage of the sensor plate and the equivalent square of the plate can be expressed as follows:

(9) $$V_{0}\left(t\right)=R_{0}\epsilon_{0}\frac{d\varphi (t)}{dt}S_{eq},$$

(10) $$S_{eq}=∯f\left(d\right)dA,$$

where $R_{0}$ is the load resistance, $\epsilon_{0}$ represents the permittivity, and $S_{eq}$ represents the equivalent square of the electrode plate. According to (9), the amplitude of the induced voltage on the electrode plate mainly depends on $S_{eq}$ and the load resistance $R_{0}$.

As for sensors with differential structures, the positive and negative electrode plates with distinct sizes are utilized to form a potential difference. The equivalent square of the positive electrode plate and negative electrode plate are denoted as $S_{eq1}$ and $S_{eq2}$, respectively. The voltage across the load resistor can be expressed as follows:

(11) $$V_{0}\left(t\right)=R_{0}\epsilon_{0}\frac{d\varphi (t)}{dt}\left(S_{eq1}-S_{eq2}\right),$$

**Figure 5:**
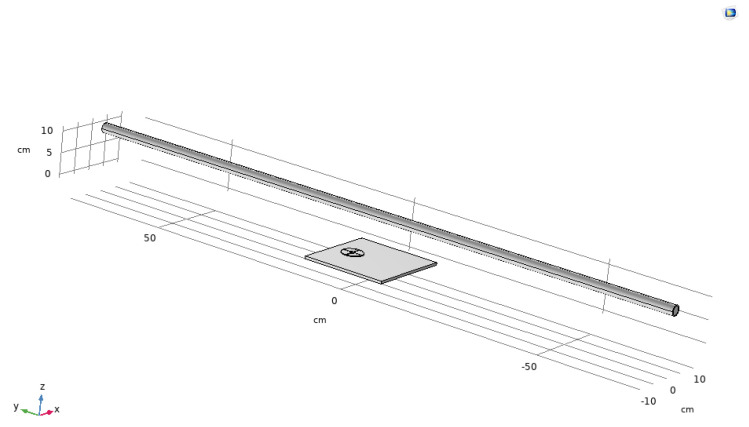
Diagram of the differential structure sensor.

The equivalent square $S_{eq}$ of the electrode plate is determined by three factors: (1) The square of the electrode plate; (2) the distance between the electrode plate and the live conductor *d*; and (3) the distance between the two plates *w*. To evaluate the influence of *d* and *w* on the sensor’s transformation ratio and output voltage amplitude, COMSOL Multiphysics is employed for simulation. The simulation scenario consists of the electrode plates and a 1 kV overhead line arranged horizontally at a height of 10 cm. The diagram of the simulation scenario is shown in Figure 5.

Firstly, we investigate the impact of *d* with a fixed w=2 cm. The simulation results are summarized in Table 1. It can be seen that the amplitude of the sensor output voltage is not sensitive to distance *d*. When the distance increases from 2 cm to 10 cm, the output voltage amplitude only decreases by about half. To meet the applicable requirements, the distance d needs to be increased to a range of 80 cm to 100 cm, which significantly raises the difficulty of sensor installation during actual measurement. Therefore, changing the distance *d* control sensor’s transformation ratio is not recommended.

Secondly, we investigate the impact of *w* with the distance *d* fixed at 2 cm. The simulation results are organized in Table 2. It can be seen that the sensor output voltage is not sensitive to the spacing *w*, and the output voltage is positively correlated with the spacing *w*. If the transformation ratio is increased, it necessitates reducing the plate spacing, which poses a challenge to the insulation design. Conversely, if the transformation ratio is decreased, the electrode plate spacing becomes excessively large, resulting in the sensor volume becoming larger and increasing the manufacturing cost.

Finally, the optimal scheme is to alter the transformation ratio by changing the square difference between the two plates of the differential structure sensor. Considering the actual installation convenience and manufacturing cost, the inner and outer radii of the positive plate are set to 5 mm and 25 mm, respectively, and the inner and outer radii of the negative plate are set to 15 mm and 25 mm, respectively. The distance *d* is 2 cm, and the distance *w* is 1.2 cm.

### 3.2. Sensor Placement

**Figure 6:**
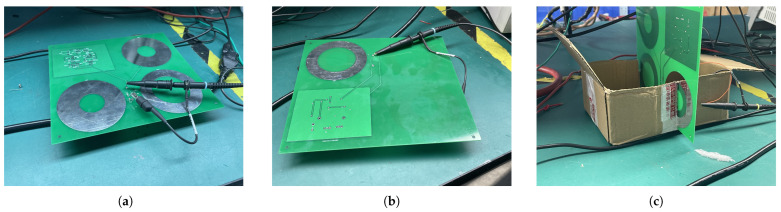
Diagram of the differential structure sensor: (**a**) positive placement; (**b**) negative placement; and (**c**) vertical placement.

The sensor can be placed in the following three ways. First, the sensor is placed parallel to the live conductor with its positive plate facing up as shown in Figure 6a, called positive placement. Second, the sensor is placed parallel to the live conductor with its negative plate facing up, which is called negative placement, as depicted in Figure 6b. Thirdly, the live conductor is equidistant from the two plates and the sensor is placed vertically to it, as shown in Figure 6c, called vertical placement. Through the experiment, 1 kV voltage is applied to the live conductor, and a 1.2/50 μs lightning surge waveform is employed to conduct high-frequency measurements on the sensor. The test results of the three placement methods are summarized in Table 3.

Through the comparative test, the following conclusions can be drawn. The falling edge of the sensor’s high-frequency response of the horizontally placed plate outperforms the vertically placed plate, while the amplitude of the induced voltage will increase; moreover, larger parallel capacitance results in the more serious deviation of the falling edge of the sensor’s high-frequency response and the smaller amplitude of the induced voltage. In addition, the parallel resistance generates little effect on the experimental results, since the voltage amplitude will be slightly increased by 5 % as the parallel resistance becomes larger. After comprehensive consideration of various factors, the positive placement configuration was ultimately selected for the electric field coupling sensor.

### 3.3. Selection of Operational Amplifier

**Figure 7:**
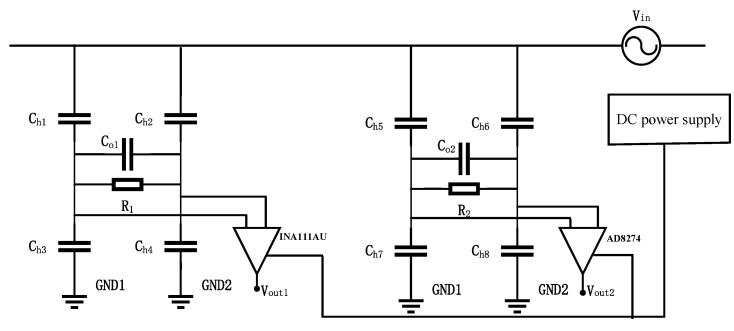
Full equivalent circuit diagram of the sensor.

In this paper, the sensor is designed with a dual channel, which consists of a high-frequency measurement channel and power-frequency measurement channel. The power-frequency measurement channel uses a INA111AU differential operational amplifier with an internal resistance of about $10^{12}$ Ω, while adopting high internal resistance will lead to the serious voltage wave reflection of the sensor’s high-frequency response, resulting in a large measurement deviation. Therefore, the high-frequency measurement channel utilizes a AD8274 differential op amp with a low internal resistance of about 36 kΩ. The whole design is shown in Figure 7.

## 4. Experimental Analysis

### 4.1. Construction of Experimental Platform

**Figure 8:**
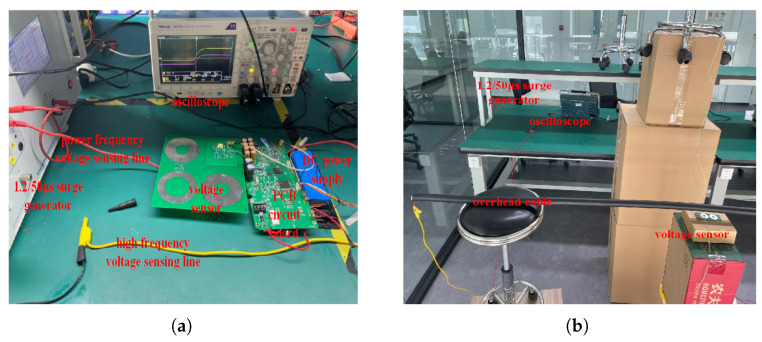

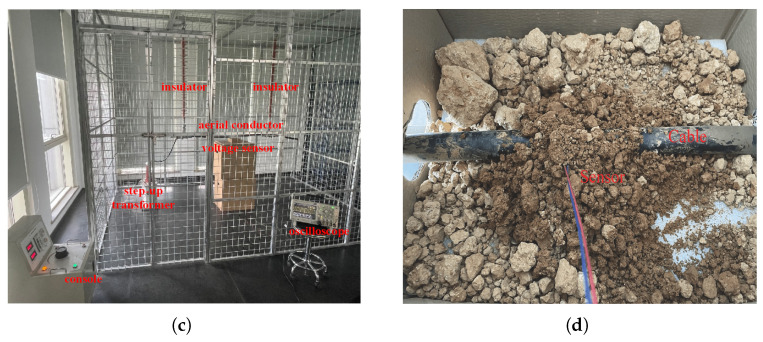
Experimental platform: (**a**) low-voltage experimental environment; (**b**) high-voltage high-frequency overhead line experimental environment; (**c**) high-voltage power-frequency overhead line experimental environment; and (**d**) high-voltage power-frequency cable experimental environment.

In this study, three experimental environments are simulated:

- Environment 1: a laboratory low-voltage insulated silicone wire experimental environment;
- Environment 2: a high-voltage overhead line experimental environment;
- Environment 3: a high-voltage cable experimental environment;

Firstly, 220 V power-frequency voltage and 1 kV surge voltage are applied to environment 1 to evaluate the optimal parameters of the sensor. Secondly, a 1.2/50 μs lightning surge generator, console, and booster transformer are used in environment 2, and the optimum sensor parameters for the power frequency and high frequency of distribution lines below 10 kV are obtained. Finally, by using the console and booster transformer, the sensor linearity in environment 2 and environment 3 are provided under the power frequency. The experimental environments are shown in Figure 8. The experimental environments mainly include the following equipment: a console, a 1.2/50 μs lightning surge generator (Model: SUG61005TB, manufactured by Shanghai Prima Electronic Co., Ltd., Shanghai, China), a step-up transformer (Model: YDJ-50/50, manufactured by Wuhan Huadian Meilun Power Technology Co., Ltd., Wuhan, China), insulators, an oscilloscope, and an overhead line. The regulation range of the console is 0∼700 V, the transformation ratio of the step-up transformer is 1:500, and the high-voltage power-frequency test environment can output voltage waves below 35 kV.

### 4.2. Influence of Parallel Resistance on Measurement

**Figure 9:**
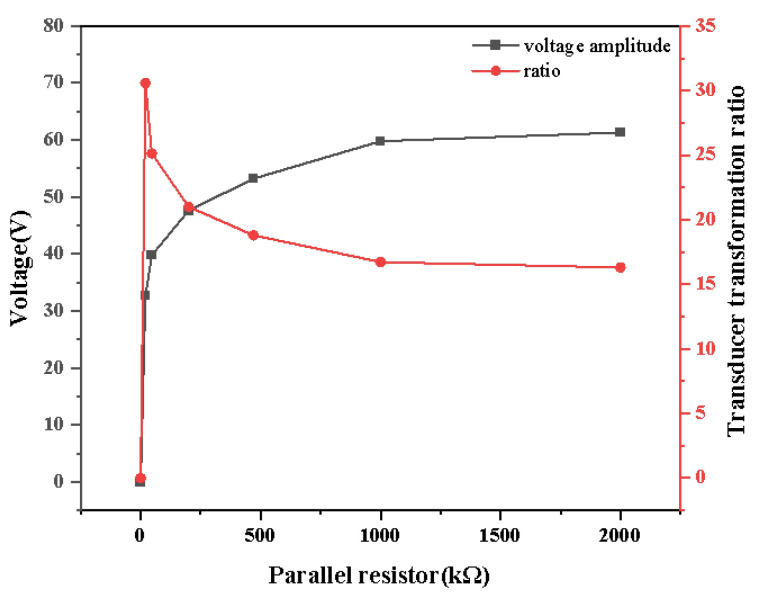
Effect of parallel resistance on voltage amplitude and transformation ratio.

Since the variation of the parallel resistor is orders of magnitude greater than the parallel capacitor, the parallel resistor predominantly determines the charge and discharge process between the plates. The parallel resistor parameters significantly affect the acquisition of high-frequency voltage signals but have little impact on the acquisition of power-frequency voltage signals. Thus, this section focuses on the influence of the parallel resistor on the sensor’s high-frequency response. First, in environment 1, a 1.2/50 μs surge generator sends out a lightning surge waveform with an amplitude of 1 kV. Without considering the parallel capacitor, the parallel resistance is adjusted only. The measured results are summed up in Figure 9. The amplitude of the sensor output signal increases continuously while the increment is diminishing. The sensor’s transformation ratio decreases with the increase in parallel resistance and eventually becomes stable. This conclusion can also be deduced from (1). Thus, the experimental results are consistent with the theoretical analysis.

**Figure 10:**
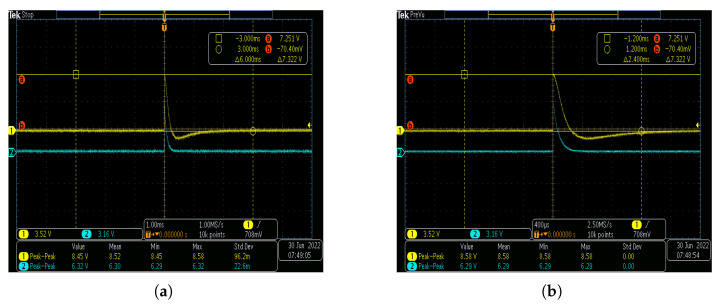
Effect of parallel resistance on high-frequency response waveform of sensor: (**a**) parallel resistance 20 kΩ; (**b**) parallel resistance 47 kΩ.

Then, the influence of resistance on the sensor waveform is analyzed. Figure 10a,b show the measured waveforms with respect to the parallel resistances of 20 kΩ and 47 kΩ, respectively. The 1.2/50 μs surge waveform has a rise time of approximately 2 μs, while the fall time (time to decay to 50% of peak) is 50 μs. The yellow line denotes the voltage waveform output by the sensor, and the blue line refers to the waveform of the original signal. Figure 10 exhibits that when the parallel resistance is 20 kΩ, the rising edge of the output waveform performs better, while the falling edge exhibits poor tracking performance. When the parallel resistance increases to 47 kΩ, the falling edge of the output waveform of the sensor is significantly improved. However, in the case of very large parallel resistance, the charging and discharging time between the plates becomes longer, which reveals that the falling edge of the sensor output waveform falls slowly, and the measurement results increasingly deviate from the original signal.

### 4.3. Influence of Parallel Capacitance on Measurement

**Figure 11:**
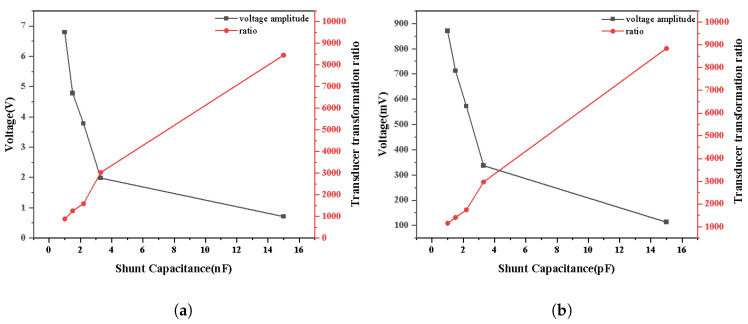
Effect of parallel capacitance on power-frequency and high-frequency signal of sensor: (**a**) power-frequency; (**b**) high frequency.

Under the overhead line power-frequency high-voltage test environment, the potential of the overhead line is raised to 6 kV through the step-up transformer, and the overhead line is in a no-load state. In the case of 1 MΩ parallel resistance, we adjust the parallel capacitance for testing. The measured results are presented in Figure 11.

Under the high-frequency experimental environment of the overhead line, 1 kV surge voltage is applied to test the high-frequency signal with 1 MΩ parallel resistance. Experimental results are displayed in Figure 11. Through Figure 11a,b, it can be concluded that the amplitude of the induced voltage is negatively correlated with the parallel capacitance and the transformation ratio is positively correlated with the parallel capacitance. When the sensor measures signals of different frequencies, its transformation ratio tends to be constant, with the increase in parallel capacitance $C_{3}$.

**Figure 12:**
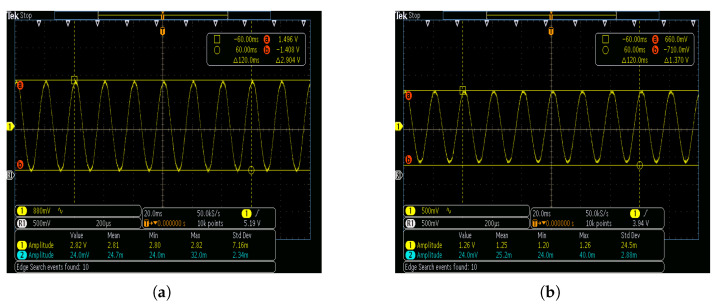
Effect of parallel capacitance on quality of power-frequency voltage waveform of the sensor: (**a**) non-parallel capacitance; (**b**) parallel capacitance.

The parallel resistance and capacitance in the middle of the plates constitute a filter circuit, which attenuates high-frequency signals and significantly enhances the waveform quality of the sensor’s power-frequency response. Under the power-frequency high-voltage experimental environment of the overhead line, the output waveform of the sensor measured by the oscilloscope is depicted in Figure 12. In the absence of the parallel capacitor, the power-frequency response waveform quality is inferior due to harmonic components, i.e., the existence of burrs in the waveform. In the presence of the parallel capacitor, its power-frequency response is more precise.

**Figure 13:**
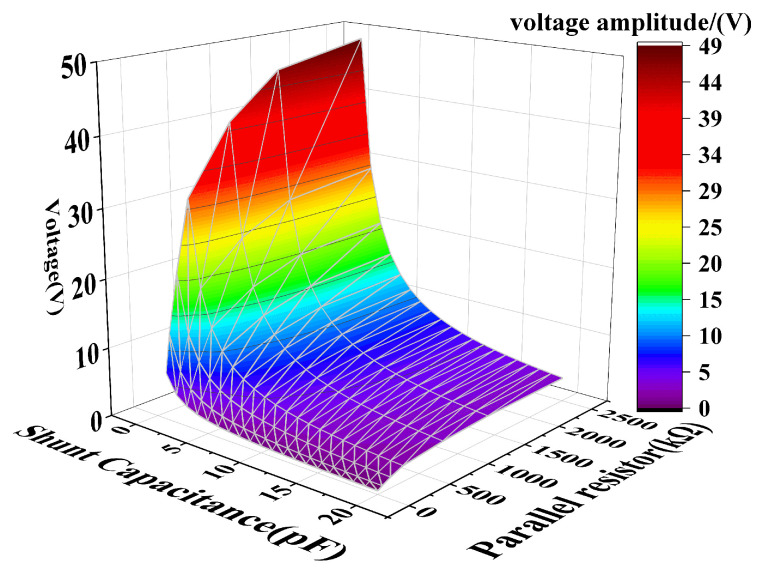
Effect of resistance and capacitance parameters on power-frequency voltage amplitude.

Through trial and error, we investigated the influence of resistance and capacitance parameters on power-frequency voltage amplitude, shown in Figure 13. Finally, the optimal parameter combination of the sensor suitable for 10 kV distribution lines is shown in Table 4.

### 4.4. Sensor Bandwidth Test

**Figure 14:**
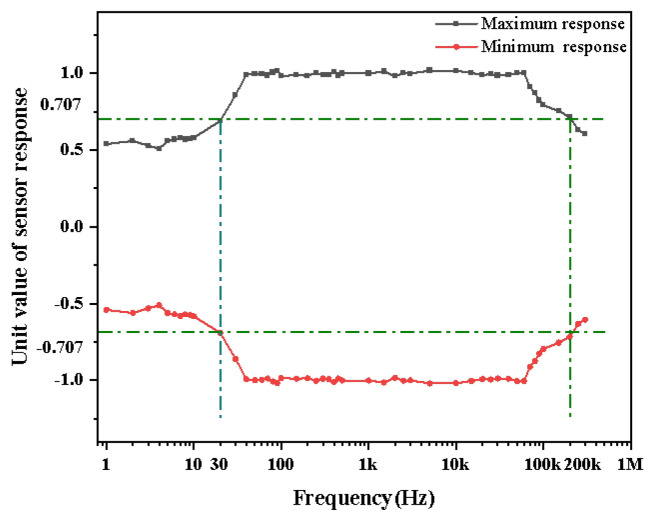
Sensor frequency response curve.

We utilize the frequency response analyzer to generate a 1 Hz–200 kHz sinusoidal voltage wave and investigate the sensor’s bandwidth. In total, 10 groups of data are measured at each frequency level, and the maximum and minimum unit values of the sensor response at each frequency are recorded, as shown in Figure 14. The response for the signal between 50 Hz and 80 kHZ is relatively stable, while the response is equal to $\sqrt{\frac{1}{2}}$ of its maximum response as the signal frequency reaches 200 kHz. To sum up, the bandwidth of the electric field coupled sensor ranges from 30 Hz to 200 kHz.

### 4.5. Sensor Linearity Analysis

**Figure 15:**
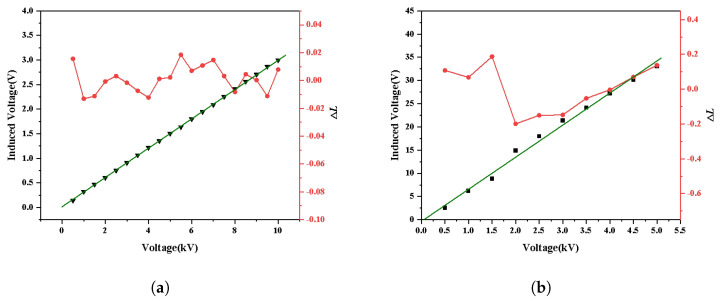
Nonlinear error performance of the sensor in power-frequency: (**a**) linear fitting results and nonlinear errors of environment 2; (**b**) linear fitting results and nonlinear errors of environment 3.

This section focuses on testing the linearity of the sensor in power frequency for environment 2 and environment 3, as shown in Figure 8c,d. Specifically, we employ the power-frequency alternating current of different voltages for testing. Firstly, the induced voltage amplitude and nonlinear error of the sensor measured in environment 2 are exhibited in Figure 15a. The induced voltage amplitude and nonlinear error of the sensor measured in the same way under the power-frequency in environment 3 are shown in Figure 15b. The nonlinear error is calculated by:

(12) $$\begin{array}{c} \delta =\frac{\left|\Delta L_{max}\right|}{Y}\cdot 100\%, \end{array}$$

where δ represents the nonlinear error, $\Delta L_{max}$ is the maximum distance from the measuring point to the fitting straight line, and *Y* denotes the maximum value of the measuring point. From Figure 15a,b, it can be seen that $\Delta L_{max}=0.0185$ and Y=2.997, and the nonlinear error of the sensor is 0.62% in environment 2. Moreover, we measured $\Delta L_{max}=0.1876$, Y=33.08, and the nonlinear error of the sensor as 0.57% in environment 3.

## 5. Conclusions

In this paper, we proposed a novel miniature non-contact voltage sensor for both power-frequency and high-frequency signals. The sensor employs a differential structure to improve measurement accuracy and reduce the influence of the environment. Specifically, we presented the basic principles of electric field coupling measurement and the transfer function of the differential structure sensor. Based on this, we derived the condition for a constant transformation ratio of the electric field sensor.

The effects of several factors on the equivalent area of the electrode plate were analyzed, and suitable parameters were determined. Through comparison, it was concluded that the optimal placement of the electrode plate is the positive placement.

Through the experiment, we investigated the effects of parallel resistors and capacitors on voltage measurement, and the optimal capacitor and resistor parameters were provided. Then, the bandwidth of the electric field coupling sensor was tested ranging from 30 Hz to 200 kHz. Through assessing the linearity of the electric field sensor in the power-frequency, the nonlinear error in the high-voltage overhead line environment was found to be 0.62%. While in the high-voltage cable environment, it was 0.57%.

## Figures and Tables

**Table 1:** Effect of *d* on sensor transformation ratio.

Potential of the Tested Conductor/kV	d/cm	Output Voltage of the Sensor/V	The Transformation Ratio of the Sensor
1	2	45.70	21.88:1
1	4	33.73	27.99:1
1	6	28.77	34.76:1
1	8	25.06	39.90:1
1	10	18.76	53.30:1

**Table 2:** Effect of *w* on sensor transformation ratio.

Potential of the Tested Conductor/kV	w/cm	Output Voltage of the Sensor/V	The Transformation Ratio of the Sensor
1	2	45.70	21.88:1
1	4	46.17	21.66:1
1	6	50.92	19.64:1
1	8	60.32	16.58:1
1	10	85.26	11.73:1

**Table 3:** Effect of parallel capacitance on sensor transformation ratio under different placement modes.

Placement	Parallel Cap- Acitance/nF	Power-Frequency Voltage Ratio	High-Frequency Voltage Ratio	Gain Ratio of High-Frequency Voltage to Power-Frequency Voltage
Positive	0	353:1	199:1	0.56:1
Positive	0.1	393:1	350:1	0.89:1
Positive	1	1234:1	1276:1	1.034:1
Positive	2.2	2287:1	2668:1	1.175:1
Negative	2.2	2990:1	4419:1	1.48:1
Vertical	4.7	33,085:1	22,727:1	0.69:1

**Table 4:** Optimal parameter combination.

Channel	Parallel Capacitance/nF	Parallel Resistor/kΩ	Ratio
power-frequency measurement	3.3	1000	300:1
High-frequency measurement	1.0	36	1200:1

## Data Availability

The original contributions presented in this study are included in the article material. Further inquiries can be directed to the corresponding author.
